# Adult Primary Central Nervous System Atypical Teratoid/Rhabdoid Tumor Metastasizing to the Cervical Lymph Node

**DOI:** 10.7759/cureus.73742

**Published:** 2024-11-15

**Authors:** Nirvana Thangjam, Biswajit Dey, Vandana Raphael, Jaya Mishra, Donboklang Lynser, Tamajyoti Ghosh, Sumit Kumar, Mainak Bhattacharjee

**Affiliations:** 1 Pathology, North Eastern Indira Gandhi Regional Institute of Health and Medical Sciences (NEIGRIHMS), Shillong, IND; 2 Radiology, North Eastern Indira Gandhi Regional Institute of Health and Medical Sciences (NEIGRIHMS), Shillong, IND; 3 Neurosurgery, North Eastern Indira Gandhi Regional Institute of Health and Medical Sciences (NEIGRIHMS), Shillong, IND; 4 Radiation Oncology, North Eastern Indira Gandhi Regional Institute of Health and Medical Sciences (NEIGRIHMS), Shillong, IND

**Keywords:** adult, atypical teratoid/rhabdoid tumor, central nervous system, cervical lymph node, metastasis

## Abstract

Atypical teratoid/rhabdoid tumors (AT/RTs) of the central nervous system (CNS) are rare and aggressive, typically occurring in early childhood or infancy, with adult cases being extremely rare. These tumors are associated with the inactivation of the integrase interactor 1 (INI1) gene. The prognosis is poor, worsening significantly if metastasis is detected at diagnosis. While CNS tumors rarely metastasize to cervical lymph nodes, recent findings have shown that such dissemination is possible, challenging the previously held belief that CNS malignancies do not spread via the lymphatic system. Awareness of this potential pathway is crucial for early diagnosis and avoiding unnecessary treatments. We present a case of a young adult male patient with a primary CNS AT/RT who had presented with cervical lymph node metastasis.

## Introduction

The term "rhabdoid tumor" was first used in 1978 by Beckwith and Palmer to describe a histological variant of Wilm's tumor in young children with a poor prognosis, chosen for its similarity to rhabdomyosarcoma despite differing muscle characteristics [1,2]. In 1985, a central nervous system (CNS) tumor primarily composed of rhabdoid cells was identified, and the term "atypical teratoid/rhabdoid tumor" (AT/RT) was coined to describe tumors with a mix of rhabdoid, primitive neuroepithelial, mesenchymal, and epithelial components [3]. AT/RTs are predominantly found in infants and young children, representing about 1-2% of all CNS tumors in children and over 10% of CNS malignancies in infants [4,5]. They are very rare in adults and more common in males under age three, with prevalence decreasing thereafter [4].

A genetic form of AT/RTs known as rhabdoid tumor predisposition syndrome, which is typically sporadic but can be inherited in an autosomal dominant manner, is caused by mutations in the integrase interactor 1 (INI1) gene (also known as SMARCB1) at 22q11.23 [4]. This genetic condition often results in cancers of the kidneys, brain, and soft tissues [4]. CNS tumors commonly spread along the craniospinal axis, with leptomeningeal spread being prevalent [6]. Extracranial metastasis is rare in CNS tumors, and only a few case reports document such spread in AT/RTs of the CNS [6,7].

We report a case of an AT/RT in the left posterior fossa of a young adult, presenting with cervical lymph node metastasis and no cerebrospinal fluid shunt.

## Case presentation

A 20-year-old male patient presented with a one-year history of swelling on the left side of his neck, with no history of fever or tuberculosis exposure. Examination revealed bilateral tonsillar enlargement and left cervical lymphadenopathy at levels Ib and V. Ultrasound showed a conglomerated lymph nodal mass (5x6x4 cm) in the left lateral neck region around stations Ib, II, and III, with a heterogeneous texture and no internal vascularity, suggesting a tubercular etiology.

A fine needle aspiration cytology (FNAC) from the cervical nodes done outside suggested a poorly differentiated malignancy. Upper gastrointestinal endoscopy revealed antral gastritis, but the rest of the study was normal. Abdominal ultrasound also showed normal results. Seeking a second opinion at our institution, the patient underwent a repeat FNAC, which showed singly scattered and occasional clusters of atypical cells among histiocytes. These cells had an increased nuclear-to-cytoplasmic ratio, coarse chromatin, and prominent nucleoli, indicating a metastatic poorly differentiated malignancy.

**Figure 1:**
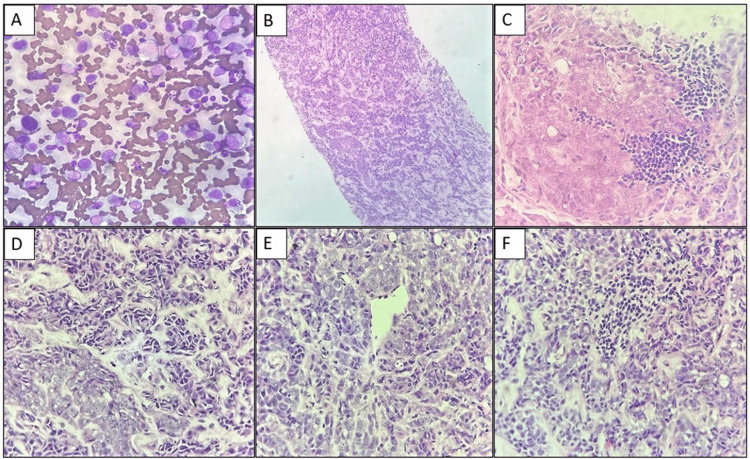
(A) Fine needle aspiration cytology smear showing enlarged atypical cells that are dispersed singly (MGG, 400x). (B) Core biopsy of lymph nodal tissue infiltrated by tumor cells (H & E, 100x). (C-F) Core biopsy of lymph node showing large tumor cells with a moderate amount of eosinophilic cytoplasm, eccentric nuclei with vesicular chromatin and prominent nucleoli (H & E, 400x) MGG: May Grunwald Giemsa stain; H & E: Hematoxylin and eosin

A biopsy of the same site yielded four grey-white linear tissue cores. Microscopy revealed small to medium-sized neoplastic cells with a high nuclear-to-cytoplasmic ratio and hyperchromatic nuclei, interspersed with some epithelioid cells. Focal areas showed large rhabdoid cells with abundant eosinophilic cytoplasm, eccentric nuclei, vesicular chromatin, and prominent nucleoli (Figure 1).

**Figure 2:**
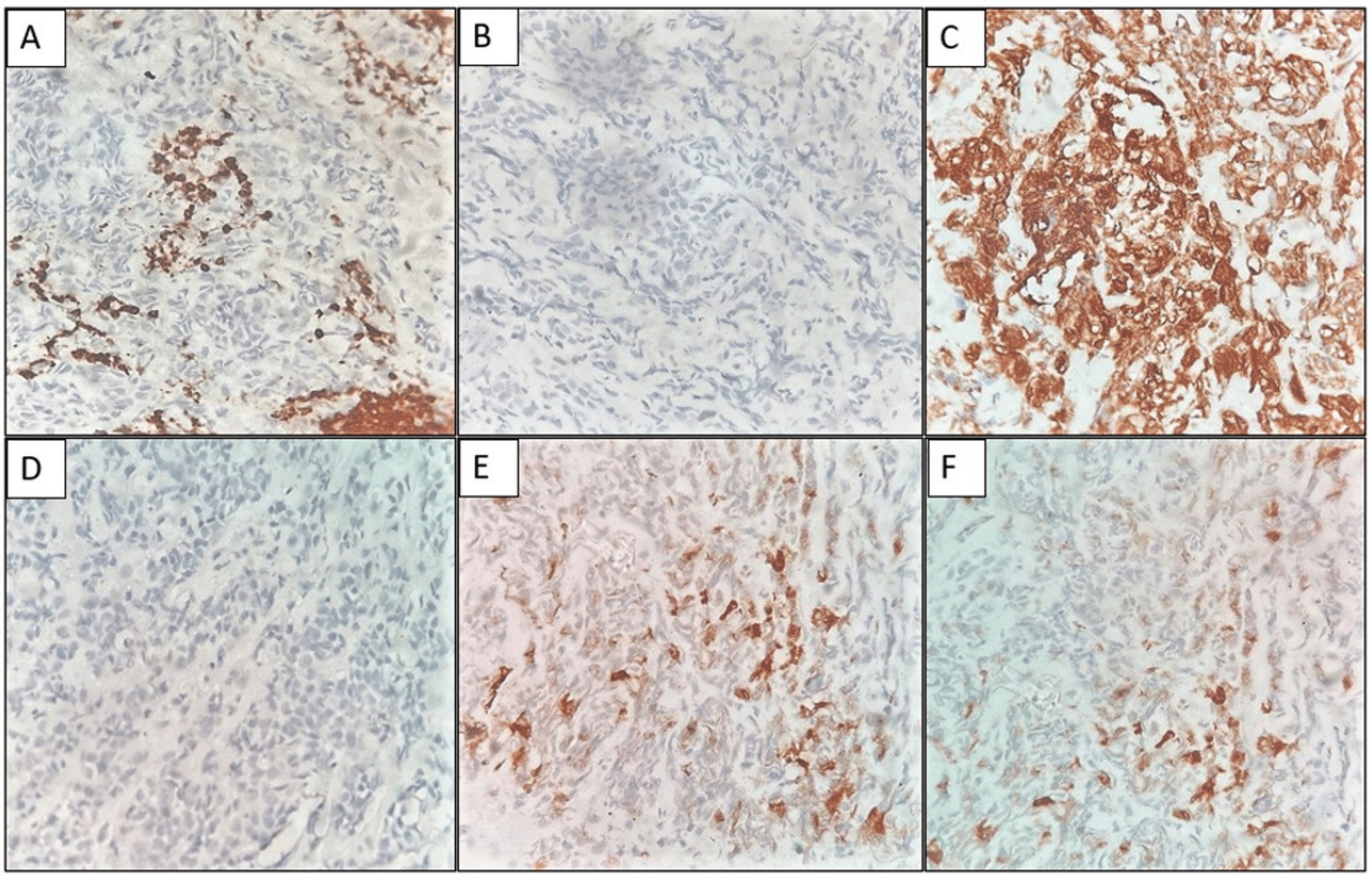
(A) LCA showing negative staining in the tumor cells and highlighting only the reactive lymphoid cells. (B) PanCK showing negative staining in the tumor cells. (C) Vimentin showing diffuse strong positive staining in the tumor cells. (D) INI1 showing loss of expression in the tumor cells. (E) EMA is positive in few of the tumor cells. (F) SMA showing focal staining in the tumor cells (IHC, 400x) LCA: Leucocyte common antigen; PanCK: Pan-cytokeratin; INI1:Integrase Interactor 1; EMA: Epithelial membrane antigen; SMA: Smooth muscle antigen; IHC: Immunohistochemistry

Immunohistochemical staining (Figure 2) was positive for vimentin, epithelial membrane antigen (EMA), and smooth muscle actin (SMA) but was negative for glial fibrillary acidic protein (GFAP), leukocyte common antigen (LCA), pan-cytokeratin (PanCK), S100, HMB445, synaptophysin, desmin, CD34, and CD30, ruling out glioma, lymphoma, carcinoma, neural tumors, malignant melanoma, neuroendocrine tumors, rhabdomyosarcoma, vascular and epithelioid sarcomas, and anaplastic large cell lymphoma. There was loss of SMARCB1/INI1 expression in the tumor cells. The magnetic resonance imaging (MRI) report was received along with the immunohistochemical stains.

**Figure 3:**
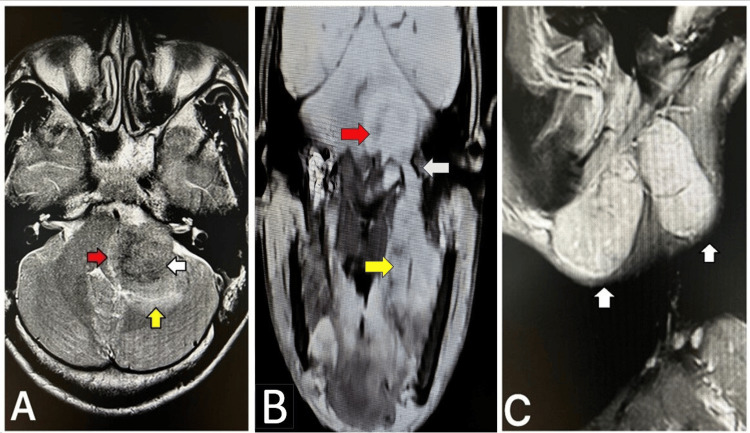
(A) T2-weighted axial image showing a left posterior fossa heterogeneous extraaxial lesion (white arrow) causing compression of the pons (red arrow) and cerebellum (yellow arrow). (B) T1FS contrast oblique-coronal image showing the enhancing left posterior fossa extraaxial lesion (red arrow) extending through the jugular foramen (white arrow) into the neck (yellow arrow). (C) T1FS contrast sagittal image showing enlarged enhancing left-sided neck lymph nodes (white arrows). T1FS: T1-weighted fat saturation

MRI of the brain and neck revealed an extra-axial lesion in the left posterior fossa, compressing the pons, left middle cerebellar peduncle, medulla oblongata, and adjacent cerebellum. The lesion extended through the jugular foramen into the left carotid space, creating a dumbbell appearance and encasing the cervical segment of the left internal carotid artery. On T1-weighted imaging, the lesion was hypointense; on T2-weighted imaging, it was heterogeneously hyperintense with a curvilinear hypointense area. The lesion showed diffusion restriction on diffusion-weighted imaging and enhancement on contrast study. Enlarged enhancing lymph nodes were noted at levels Ib and V, with the largest measuring 5.4×3.2×2.9 cm at level Ib. These lymph nodes also showed diffusion restriction and enhancement in the post-contrast study (Figure 3). MRI further revealed medialization and thickening of the left aryepiglottic fold, suggesting left vocal cord palsy, and bilaterally enlarged palatine tonsils. A cytological examination of cerebrospinal fluid revealed no malignant cells.

Based on the morphology, immunohistochemistry, and radiological features, the final diagnosis of a metastatic atypical teratoid/rhabdoid tumor originating from the primary central nervous system was made. Surgery was not possible as the lesion was encasing the internal carotid artery. The patient received palliative radiotherapy of 30 grays in 10 fractions targeting the whole brain and metastatic nodes, resulting in symptomatic improvement. Palliative chemotherapy was planned, but the patient refused further treatment and was lost to follow-up.

## Discussion

The AT/RT is a rare and aggressive embryonal CNS tumor. The "atypical" refers to its teratoid component, which has features of two germ cell layers but differs from classic teratoid tumors [8]. "Rhabdoid" describes the tumor's rod-like appearance, resembling skeletal muscle differentiation. This case involves an AT/RT in the left posterior fossa, an unusual location for this tumor. Differential diagnoses include the rhabdoid variant of meningioma, epithelioid glioblastoma with a rhabdoid component, rhabdoid chordoma, and, very rarely, epithelioid sarcoma [8,9].

Given the numerous differential diagnoses, immunohistochemistry is paramount for diagnosing AT/RTs. These tumors typically show positivity for rhabdoid cell markers such as vimentin, EMA, and SMA. In contrast, markers specific to germ-cell tumors like placental alkaline phosphatase and alpha-fetoprotein are negative [4]. Additionally, neurofilament protein, PanCK, synaptophysin, and glial fibrillary acidic protein may be expressed [4]. AT/RT-specific immunohistochemistry staining for the INI1 protein, part of the SWI/SNF chromatin-remodeling complex, is highly sensitive and specific [4]. In this case, immunohistochemistry markers, along with morphological and radiological features, were essential for diagnosing AT/RTs from the metastatic cervical lymph node biopsy.

Extracranial metastases from CNS malignancies often originate from head and neck cancers but can also come from sites like the lungs, breast, digestive, or reproductive systems, usually affecting lower neck nodes [10]. While the general incidence ranges from 0.4% to 2.3%, some studies report rates of 10% to 20% [6]. Although rare, cervical lymph node metastases from primary CNS tumors should be considered when diagnosing unknown primary malignancies.

For patients with a history of CNS tumors, craniotomy, or cranial irradiation, pathologists should consider the possibility of CNS malignancy metastasizing to cervical lymph nodes [11]. While malignant CNS tumors rarely spread outside the CNS, when they do, they most commonly metastasize to the lungs, pleura, lymph nodes, liver, kidneys, and bone, with bone being the most frequent site [12]. Cervical lymph nodes are the most common lymphatic sites for such metastases [6]. The rarity of extracranial spread is attributed to the blood-brain barrier and the limited presence of lymphatic capillaries in the CNS [10].

Extracranial metastasis from primary CNS tumors without prior surgery is rare [12]. In this case, there was no history of surgery. One pathway for such metastasis is the intracranial lymphatics, which connect to cervical lymph nodes via the jugular foramen [10]. Although a few cases of extracranial metastasis in CNS AT/RTs exist, there are no published reports of cervical lymph node metastasis of AT/RTs in the scientific literature in English language. The prognosis for AT/RTs is generally poor, worsening significantly if metastasis is present at diagnosis, which complicates treatment and reduces the chances of a successful outcome.

Patients with AT/RTs often receive additional treatments post-surgery, including radiation, chemotherapy, targeted therapy, or immunotherapy. Surgery is considered if the patient’s health allows it and if the neck masses are removable. The primary tumor's operability and the distant metastases are also important factors [10]. In this case, standardizing treatment was challenging due to the rarity of cervical lymphatic metastases from CNS malignancies. Palliative radiotherapy followed by chemotherapy was planned, as surgical resection of the primary tumor was not feasible due to its invasive nature.

## Conclusions

AT/RTs are rare and highly aggressive embryonal CNS tumors characterized by their distinctive teratoid and rhabdoid features. Extracranial metastasis is rarely reported and cervical lymph node metastasis is a rare metastatic site in CNS tumors, which confers a poor prognosis. Differential diagnoses include other rhabdoid and epithelioid tumors, necessitating precise immunohistochemical profiling to confirm AT/RTs. Treatment standardization is challenging in these cases. This case is notable due to the tumor's unusual location in the left posterior fossa and its metastatic spread to cervical lymph nodes, a rare occurrence for CNS malignancies.
